# CD44^+^ gastric cancer cells with stemness properties are chemoradioresistant and highly invasive

**DOI:** 10.3892/ol.2013.1272

**Published:** 2013-03-26

**Authors:** MAO SUN, WEI ZHOU, YUN-YUN ZHANG, DONG-LIN WANG, XIAO-LING WU

**Affiliations:** 1 Department of Gastroenterology, The Second Affiliated Hospital of Chongqing Medical University, Chongqing 400010;; 2 Departments of Radiology, Chongqing Cancer Hospital, Chongqing 400011. P.R. China; 3 Oncology, Chongqing Cancer Hospital, Chongqing 400011. P.R. China

**Keywords:** CD44, gastric cancer, stem cell, chemoradioresistance, cancer invasion

## Abstract

CD44 has been confirmed as a cancer stem cell marker in a variety of human cancer cell lines and primary tumours, but whether this marker is applicable to gastric cancer (GC) remains unknown. The responses of CD44^+^ GC stem-like cells to chemoradiation and the roles they play in cancer invasion are not well understood. In the present study, cell sorting was applied to the poorly differentiated human GC cells to isolate a pure concentration of the CD44^+^ cell populations (<1% CD44^−^ cells). The stemness properties of the CD44^+^ cell population were confirmed by two ‘gold standard’ methods; an *in vivo* tumourigenicity assay and an *in vitro* spheroid colony formation assay. In addition, the treatment response was evaluated in CD44^+^ and CD44^−^ cell fractions that underwent chemoradiation. In general, CD44^+^ stem-like cells tended to respond more poorly to chemoradiation than their non-stem-like counterparts. Further experimentation revealed that the CD44^+^ stem-like cells that recorded positive scores in the migration and invasion assay *in vitro* formed invasive tumours *in vivo*. Therefore, we hypothesized that CD44^+^ stem-like cells may significantly express invasion-associated genes. Consistent with this prediction, increased expression of the cancer invasion-related genes matrix metalloproteinase (MMP)-1, MMP-2, epidermal growth factor receptor (EGFR) and cyclooxygenase 2 (COX-2) were detected in the CD44^+^ stem-like cells. To the best of our knowledge, this is the first study that reveals the correlation between CD44^+^ GC cells and cancer invasion. By selectively eliminating CD44^+^ stem-like cells, it may be possible to treat patients with aggressive, non-resectable GCs, as well as preventing the tumours from metastasizing.

## Introduction

Gastric cancer (GC) is the fourth most common type of cancer (989,000 cases, 7.8% of the total) and the second highest cause of cancer mortality worldwide (738,000 mortalities, 9.7% of the total) (1). GC rates have decreased noticeably over the majority of the world, but remain a burden to Eastern Asian countries (mainly China) (2). By the time symptoms occur, the cancer has often reached an advanced stage and may have also metastasized.

Clinicians are able to treat patients with appropriate doses of chemoradiotherapy and schedules to achieve logarithmic cancer cell death, while unavoidably killing normal cells that undergo rapid division (3). Due to advances in the understanding of the molecular and cellular basis of cancer, current therapeutic strategies focus on inhibiting the molecular drivers of cancer. What all these strategies, whether from the past or present, have in common is that they treat cancer as a homogeneous, abnormal entity. Therefore, drugs targeting molecular lesions should be equally effective against all tumour cells, barring the emergence of a resistant subpopulation (4).

Cancer cells are heterogeneous, not only in morphology, but also in functionality (i.e., marker expression, proliferation capacity and tumourigenicity). Only a minority of tumour cells, termed cancer stem cells (CSCs), have the capacity to regenerate the tumour and sustain its growth when injected into immune-compromised mice (5). Based on accumulating evidence, the American Association for Cancer Research (AACR) made a consensus definition of CSCs in 2006 as ‘cells within a tumour that possess the capacity for self-renewal and that can cause the heterogeneous lineages of cancer cells that constitute the tumour’ (6).

Studies of gastric CSCs (GCSCs) began relatively late compared with other solid tumours. In 2009, Takaishi *et al*(7) screened a series of potential stem cell markers in various human GC cell lines and demonstrated for the first time that CD44 may be an appropriate marker for stem cells. However, similar studies from clinical research are rare. To date, the theory of CSCs as a subpopulation with chemo/radioresistance has been verified in a number of solid tumours with the exception of GC (8–12). Furthermore, the role that GC stem-like cells play in cancer invasion remains to be elucidated.

## Materials and methods

### Cells and animals

Poorly differentiated human GC cells were derived from a 55-year-old female patient, who provided written informed consent. The patient did not undergo chemoradiotherapy prior to resection. A total of 30, 4-week-old Balb/cA nu/nu female mice were obtained from the Shanghai Experimental Animal Centre of the Chinese Academy of Science (Shanghai, China). The mice were maintained in plastic cages (five mice/cage) in a room with a constant temperature (22±1°C) and a dark-light cycle (12 h/12 h). The animal experiments and human research were performed in accordance with the ethics code approved by the Ethics Committee of Chongqing Medical University, Chongqing, China.

### Fluorescence-activated cell sorting (FACS)

For the FACS, 80% confluent cells in a 100-mm cell plate (5–10 million cells per plate) were harvested and incubated for 30 min at room temperature, with a 10-fold dilution of the following antibodies: Anti-CD44-fluorescein isothiocyanate rat monoclonal antibody and anti-CD44-PE (eBioscience, San Diego, CA, USA). The cells were then detected using a FACS-LSRII flow cytometer (Becton Dickinson, Franklin Lakes, NJ, USA).

### Spheroid colony formation assay

The CD44^+^ and the CD44^−^ fractions from the FACS-sorted GC cells were inoculated into each well (20 cells per well) of the ultra-low-attachment 48-well plates and supplemented with 300 *μ*l Dulbecco’s modified Eagle’s medium (DMEM) plus 40 ng/ml basic fibroblast growth factor (bFGF) and 20 ng/ml epidermal growth factor (EGF; Invitrogen, Carlsbad, CA, USA). After 4 weeks, each well was examined under a light microscope (Olympus, Tokyo, Japan) and the total wells with spheroid colonies were counted. Each experiment was performed at least three times.

### In vivo xenograft assay

The CD44^+^ and CD44^−^ fractions were maintained in sterile DMEM supplemented with 10% fetal bovine serum (FBS). Both cell fractions were left to grow to be tested for tumourigegnicity. The cells were harvested when they were subconfluent and adjusted to the concentration of the cell suspension to be inoculated to 5×10^4^/ml (CD44^+^) and 5×10^6^/ml (CD44^−^) in PBS. A total of 0.2 ml of the cell suspension was subcutaneously injected into the left (CD44^−^) and right (CD44^+^) hind limbs of the mice. The mice were observed daily and inspected for tumour growth each week for 6 weeks.

### In vitro cell migration and invasion assay

Matrigel was diluted in serum-free DMEM (5 mg/ml), then 100 *μ*l of this was introduced into the upper chamber of a 24-well millicell with a 8 *μ*m pore size insert (Millipore, Billerica, MA, USA). The cells were harvested and resuspended at a density of 1×10^4^/ml in media containing 0.1% FBS. Next, 100 *μ*l of the cell suspension was introduced onto the matrigel. The lower chamber of the millicell was filled with 600 *μ*l DMEM containing 10% FBS. The cells on the lower side of the insert filter were then stained with a 1% crystal violet solution for 20 min. The average number of stained cells was counted on the lower side of the filter using an inverted wide-field microscope (Olympus, Tokyo, Japan). Each experiment was performed at least three times.

### Cell treatment

The anti-cancer drug, 5-fluorouracil (5-FU; Sigma-Aldrich, St. Loius, MO, USA), was used to assess the responses of the sorted cells. To evaluate the cell viability, the sorted cells were seeded in a flat-bottomed microculture 96-well plate (2,000 cells/well) and allowed to adhere for 24 h. The cells were then treated with 5-FU (10 nM; 30 *μ*M) in phenol-free DMEM medium for 72 h. To measure the reactive oxygen species (ROS) accumulation, the sorted cells were plated in 6-well plates and stimulated with 5-FU (50 *μ*M).

### MTT assay

Once the sorted cells had been treated with 5-FU for 72 h, MTT was added to a final concentration of 0.5 mg/ml and the cells were incubated for 4 h at 37°C. The culture medium was then removed and the remaining blue precipitate was solubilized in dimethyl sulfoxide (DMSO). The absorbance at 570 nm was read using a microplate reader (BioTek, Winooski, VT, USA). This reading was divided by the adjusted absorbance reading of the untreated cells in the control wells. The IC_50_ was calculated by non-linear regression analysis using sigmoidal fitting from the sigmoidal dose-response curve. For each concentration of 5-FU, five wells were analysed. Each experiment was performed at least three times.

### ROS assay

A ROS assay kit was purchased from the Beyotime Institute of Biotechnology (Haimen, Jiangsu, China) and used according to the manufacturer’s instructions. Briefly, to load the probe *in situ*, 2 ml 2′,7′-dichlorofluorescin-diacetate (DCFH-DA; 1:1000) fluorescent probe diluted with serum-free DMEM was loaded onto each well of the 6-well plate. The plates were incubated at 37°C for 20 min and then washed with serum-free media three times. The cells were stimulated with 5-FU (50 *μ*M) for 2 h. Following this stimulation, dichlorofluorescein (DCF; excitation 488/emission 525) fluorescence was assessed immediately by flow cytometry.

### Irradiation

For the colony forming assays, the sorted cells were irradiated using a Varian Clinac iX linear accelerator (Varian, Palo Alto, CA, USA) at a dose rate of 3 Gy/min for the time required to generate dose curves of 0, 2, 4, 6 and 8 Gy. The corresponding control was sham irradiated. Colony forming assays were performed immediately after irradiation by plating the cells into 6-well culture plates. After 20 days, the colonies containing >50 cells were counted. A radiation survival curve was generated using Albright’s method (13). For the comet assays, the sorted cells were irradiated, using the method previously stated, with 0, 2 and 4 Gy. The performance of the comet assay was mainly based on the method described by Olive *et al*(14). A total of 30 representative cells were investigated per slide. The ‘comets’ were measured using the image analysis Comet Assay Software Project (CASP; Free Software Foundation, Inc., Boston, MA, USA). The Olive Tail Moment (OTM), used to quantify DNA damage, was calculated as follows: Median DNA migration distance × relative amount of DNA in the tail of the comet.

### Real-time PCR

Quantitative real-time RT-PCR was performed using a 2X Maxima SYBR Green/ROX qPCR Master mix (Thermo Fisher Scientific, Co., Ltd., Milford, MA, USA). Reactions were carried out using iCycler (Bio-Rad, Hercules, CA, USA) and the results were evaluated using the iCycler real-time detection system software. Relative quantitation of target gene expression was evaluated using the comparative Ct method.

### Western blot

The immunoreagents used for the western blot analysis were rabbit monoclonal antibodies against matrix metalloproteinase-1 (MMP-1) and cyclooxygenase 2 (COX-2) (1:100) and mouse monoclonal antibodies against MMP-2 and epidermal growth factor receptor (EGFR; 1:100). Mouse polyclonal anti-actin antibodies (1:1000; Santa Cruz Biotechnology, Inc., Santa Cruz, CA, USA) were used as the loading control. The blots were developed using a standard enhanced chemiluminescence (ECL) method (Pierce Biotechnology, Inc., Rockford, IL, USA).

### Immunofluorescence staining

CD44^+^ cell spheroids were fixed and blocked in PBS solution with 10% FBS. The primary antibodies (rabbit anti-MMP-1 and mouse anti-MMP-2; 1:100) were incubated at 4°C overnight. The fluorescent secondary antibodies [goat anti-rabbit IgG antibodies conjugated with fluorescein isothiocyanate (FITC) and goat anti-mouse IgG antibodies conjugated with tetramethylrhodamine isothiocyanate (TRITC); 1:100] were added and incubated at 37°C for 30 min. In the negative controls, the primary antibodies were substituted with PBS. The cell nuclei were counter-stained with 4′,6-diamidino-2-phenylindole (DAPI). Fluorescence was observed under a laser scan confocal microscope (Leica Microsystems, Wetzlar, Germany).

### Histological examination

Tumour tissues were fixed in 10% neutral-buffered formalin, embedded in paraffin, sectioned and stained with haematoxylin and eosin (HE). The histological differences were examined under microscopy at magnification ×40.

### Statistical analysis

Data are presented as the mean ± standard error of the mean (SEM). GraphPad Prism 5.0 software (GraphPad Software, Inc., San Diego, CA, USA) was used for statistical analysis. Statistical differences in the IC_50_ were determined using the F test. The remaining comparisons between the groups were evaluated using an unpaired t-test. P≤0.05 was considered to indicate a statistically significant difference.

## Results

### Confirmation of stemness properties of CD44^+^cells

CSCs have been shown to form suspended cell spheroids under rich growth factor, low-attachment and serum-free culture conditions (15). In the spheroid colony formation assay, the CD44^+^ cells formed more spheroids than the CD44^−^ cells (P<0.01) (Table I). The diameter of the CD44^+^ spheroids reached ∼120 *μ*m, containing ∼1,000 cells (data not shown). The tumourigenic capacity of the CSCs varied between 10- and 100-fold compared with that of the non-stem-like counterparts in different cancer cells (16–18). In the tumourigenicity assay, individual difference variables of the nude mice were excluded by an injection of the two cell fractions in the same mouse. In general, an injection of 1×10^4^ CD44^+^ cells gave rise to tumours with 80% incidence, with relatively short latency periods (<1 week) in all mice. In contrast, an injection of 1×10^6^ CD44^−^ cells conferred a tumour formation with a very low engraftment rate of 27% (P<0.01) (Table I). This may be as the CD44^+^ cells exhibited the correct intrinsic properties to form the tumours.

### Responses of CD44^+^ stem-like cells to chemoradiation

**Figure 1:**
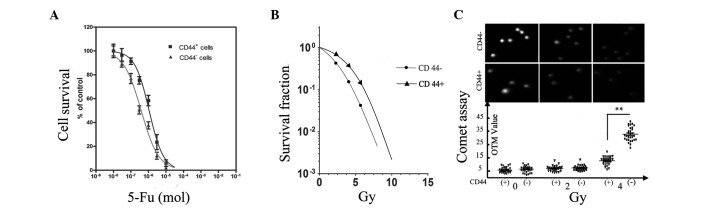
Responses of CD44^+^ and CD44^−^ GC cells to chemoradiation. (A) The IC_50_ of 5-FU was calculated by non-linear regression analysis using sigmoidal fitting from the sigmoidal dose-response curve. (B) Sorted cells were irradiated with a linear accelerator at a dose rate of 3 Gy/min for the time required to generate dose curves of 0, 2, 4, 6 and 8 Gy. (C) The two cell fractions were irradiated with 0, 2 and 4 Gy. DNA damage was quantified by measuring the OTM, n=30. Statistically significant differences are indicated as ^**^P<0.01. GC, gastric cancer; 5-FU, 5-fluorouracil; OTM, Olive tail moment.

In an MTT assay based on the calculated IC_50_ values, the responses of the two cell fractions to 5-FU varied significantly. The log IC_50_ of the CD44^+^ and CD44^−^ cells was −5.961±0.04566 and −6.415±0.04231, respectively (P<0.05; Fig. 1A). A ROS assay was performed to explore the underlying mechanism of chemoresistance. Fluorescence enhancement was consequently observed in the two cell fractions. However, the intracellular fluorescence value in the CD44^+^ cells was considerably lower than that of the CD44^−^ cells (P<0.05; Table I).

Cytotoxic oxidative stress resulting from ROS may damage DNA, RNA, proteins and lipid components, which may lead to cell death. Most significantly, ROS-induced cell apoptosis occurs in the early stages in response to chemotherapeutic drug treatment (19). Therefore, we hypothesized that the reduction in oxidatively-generated DNA damage in the CD44^+^ cells may be due to their antioxidant ability. Clonogenic and comet assays were conducted to independently evaluate the proliferative death of the cells and to quantify the DNA breaks. Notably, the radiation survival curve of the two cell fractions had a shoulder that was characterized by a comparably higher resistance at lower doses of radiation. The curve shoulder of the CD44^−^ cells disappeared at survival fraction (SF) 4 Gy. In contrast, the shoulder existed in the CD44^+^ stem-like cells until the radiation dose increased to 6 Gy (Fig. 1B). In the comet assay, irradiated cells with damaged DNA fragments formed a tail around the DNA head following electrophoresis. The OTM value was positively correlated with the extent of the damaged DNA. Consistent with the clonogenic assay, the difference in the OTM value was not significant when irradiated with 2 Gy (P>0.05). In contrast, the difference became considerably larger when the irradiation doses increased to 4 Gy (P<0.01; Fig. 1C).

### Enhancement of invasion capacity of CD44^+^ stem-like cells

**Figure 2:**
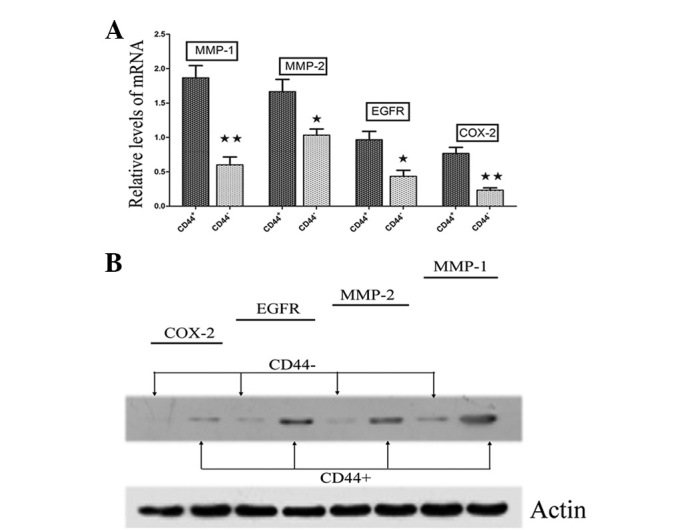
Upregulation of cancer invasion-related genes in CD44^+^ GC cells. (A) Expression of mRNA of cancer invasion-related genes; MMP-1, MMP-2, EGFR and COX-2. Results are presented as relative gene expression, where mRNA levels were calculated using the equation 2^−ΔCt^ (difference in Ct between β-actin and the target gene). Statistically significant differences are indicated as ^*^P<0.05 and ^**^P<0.01. (B) Expression of MMP-1, MMP-2, EGFR and COX-2 proteins was detected by western blot analysis. β-actin was used as the loading control in all experiments. GC, gastric cancer; COX-2, cyclooxygenase 2; MMP, matrix metalloproteinase; EGFR, epidermal growth factor receptor.

**Figure 3:**
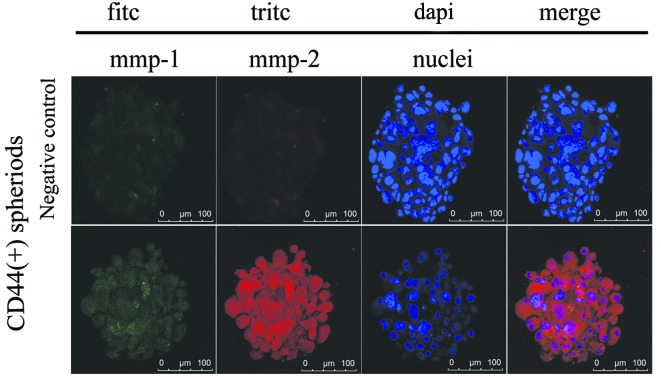
Co-expression of MMP-1 and MMP-2 in CD44^+^ GC cell spheroids. Representative immunofluorescence staining showing CD44^+^ cell spheroids, MMP-1 (green); MMP-2 (red); co-localization of MMP-1 and MMP-2 (merged). MMP, matrix metalloproteinase; GC, gastric cancer; Fitc, fluorescein isothiocyanate; tritc, tetramethylrhodamine isothiocyanate; dapi, 4′,6-diamidino-2-phenylindole.

The invasive and migratory capacities of the CD44^+^ stem-like cells were much higher compared with the CD44^−^ cells (P<0.01; Table I). The CD44^+^ cells were able to penetrate into and pass through the matrix *in vitro,* which indicated that they may synthesize more MMPs. A previous study revealed four important cancer invasion-related genes, MMP-1, MMP-2, EGFR and COX-2, in a variety of human cancers, including GC, which may manipulate the migration of tumour cells and the formation of new tumours by facilitating the release of tumour cells into the circulation (20). Therefore, the present study compared the expression profiles of the four cancer invasion-related genes in the CD44^+^ stem-like cells and their non-stem-like counterparts. This process contributed to the validation of the four genes, showing considerably varied RNA (Fig. 2A) and protein (Fig. 2B) expression levels. Furthermore, immunofluorescence staining determined that two representative genes, MMP-1 and MMP-2, were co-expressed in the CD44^+^ cell spheroids. Strong immunoreactivity was detected for each of the proteins localized in the whole cancer cells (Fig. 3).

**Figure 4:**
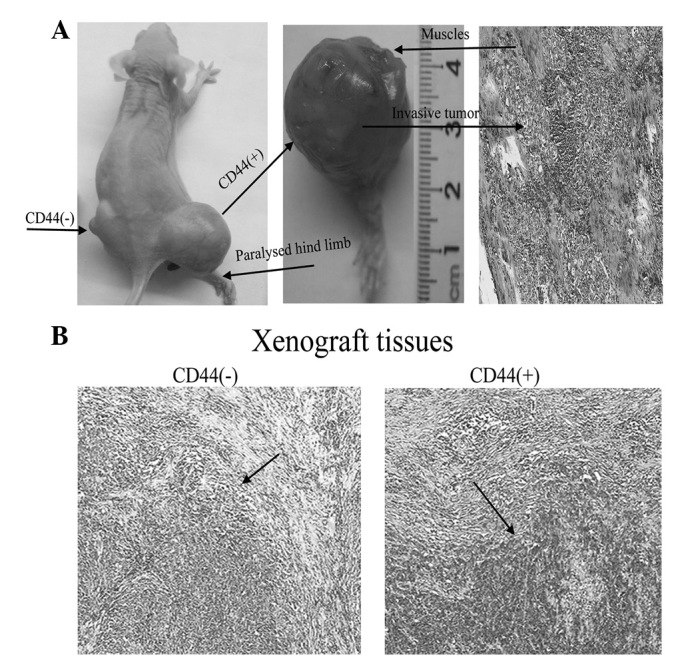
Enhancement of invasion capacity of CD44^+^ GC cells. All samples were collected, formalin-fixed and stained with haematoxylin and eosin (HE). Magnification, ×40. (A) A representative mouse with progressive muscle invasion by a tumour was paralysed in its hind limb. HE histological staining of the mass removed from the hind leg of the mouse, demonstrating invasive cancer within the muscle, consistent with poorly differentiated adenocarcinoma (right). (B) Histological differences between the CD44^+^ and CD44^−^ tumours (black arrows). GC, gastric cancer.

The CD44^+^ GC stem-like cells that recorded a positive score in the migration and invasion assay *in vitro* formed invasive tumours *in vivo*. Fig. 4A shows a representative mouse with progressive muscle invasion by a tumour that paralysed its hind limb. HE histological staining of the mass identified an invasive tumour within the muscle that was consistent with a poorly differentiated adenocarcinoma. Moreover, histological differences were observed between the CD44^+^ and CD44^−^ tumours. The CD44^+^ tumours with larger, irregular and hyperchromatic nuclei were more heterogeneous (Fig. 4B).

## Discussion

Numerous studies have particularly focussed on the signalling pathways that may mediate the resistance of CSCs (21,22). The present study contributes to the understanding of the antioxidant ability of CSCs as another important mechanism in relation to chemoradioresistance. The expression of CD44 in GC cells enhances tumourigenicity. This observation is in accordance with the significance of CD44^+^ cancer cells in the tumourigenicity of other cancers (7,23–25). The most unique and significant observation of the present study was the increased invasion capacity of the CD44^+^ stem-like cells *in vivo* and *in vitro*. Furthermore, the present study verified the expression of the cancer invasion-related genes, MMP-1, MMP-2, EGFR and COX-2, which were upregulated in the CD44^+^ GC cells, indicating a capacity to manipulate cancer invasion and even metastasis.

These observations may have clinical implications. An examination of the CD44^+^ cells in primary GC may predict the development of distant metastasis. This may facilitate patient selection for adjuvant chemoradiotherapy to reduce the chance of recurrence following resection of primary GC. During the write up of the present study, another study group successfully isolated GCSCs from the peripheral blood of cancer patients using CD44 surface markers (26). This demonstrated that GCSCs have the ability to be transferred to any of the organs of the body through the blood circulation. In total, ∼10^6^ cancer cells per gram of cancer tissue are shed into the bloodstream daily (27). However, this is not a reflection of the amount of distant metastasis found. Most cancer patients, even in their late-stage, still have very few distant metastasis. Thus, we speculated that only a small minority of cancer cells with a metastatic capacity (i.e., the CSCs) are responsible for cancer invasiveness and metastasis.

At least two important features of CD44 make it a suitable CSC marker. CD44 is a receptor for hyaluronic acid and is also able to interact with other ligands, including collagens and MMPs (28). Therefore, the CSCs that express CD44 may manipulate cell invasion, migration and adhesion to the matrix (29). Furthermore, CD44^+^ cancer cells are considered to be slow-cycling, therefore insensitive to chemoradiotherapies (30). The present study also observed that sequentially-irradiated GC cells with low X-ray doses resulted in the inhibition of DNA synthesis and the accumulation of CD44^+^ GC cells in the G_0_/G_1_ phase of the cell cycle (data not shown). This revealed that the cell cycle of CD44^+^ GC cells may be effectively regulated to avoid DNA damage by external stimuli.

## Figures and Tables

**Table I:** Comparative analysis of CD44^+^ and CD44^−^ GC cells *in vivo* and *in vitro.*

Assay	CD44^+^ GC cells	CD44^−^ GC cells	P-value
Spheriod colony formation	26.33±2.906 (n=3)	8.667±1.453 (n=3)	0.0056
Tumourigenic capacity	4.000±0.258 (n=6)	1.500±0.223 (n=6)	<0.0001
Matrigel invasion assay	74.33±8.988 (n=3)	22.00±5.508 (n=3)	0.0077
ROS (intracellular fluorescence value)	58.67±3.930 (n=3)	82.00±3.512 (n=3)	0.0114

GC, gastric cancer; ROS, reactive oxygen species.
